# *BIRC5* Genomic Copy Number Variation in Early-Onset Breast Cancer

**DOI:** 10.7508/ibj.2016.04.009

**Published:** 2016

**Authors:** Kimia Ghaffari, Mehrdad Hashemi, Elmira Ebrahimi, Reza Shirkoohi

**Affiliations:** 1 Department of Genetics, Tehran Medical Sciences Branch, Islamic Azad University, Tehran, Iran; 2 Group of Genetics, Cancer Research Center, Cancer Institute of Iran, Tehran of Medical Sciences, Tehran, Iran

**Keywords:** BIRC5, amplification, Breast cancer, Amplification

## Abstract

**Background:**

Baculoviral inhibitor of apoptosis repeat-containing 5 (*BIRC5*) gene is an inhibitor of apoptosis that expresses in human embryonic tissues but it is absent in most healthy adult tissues. The copy number of *BIRC5* has been indicated to be highly increased in tumor tissues; however, its association with the age of onset in breast cancer is not well understood.

**Methods:**

Forty tumor tissues of breast cancer were obtained from Tumor Bank of Cancer Institute, Imam Khomeini Hospital, Tehran, Iran. *BIRC5* gene copy number variation (CNV) was evaluated using Multiplex Ligation-dependent Probe Amplification (MLPA) and then compared with the age of onset for each patient.

**Results:**

*BIRC5* amplification was seen in 17.5% of cases. Also, a significant association was observed between *BIRC5* gene amplification and individuals under 40 years of age (*P*=0.04).

**Conclusion:**

*BIRC5* gene has the potential to be a marker for the detection and prognosis of cancer at an early age.

## INTRODUCTION

Breast cancer is an important health problem worldwide, and it is the second most common cancer among populations in both developed and developing countries. In Iran, breast cancer comprises 21.4% of female cancers[1].

There are several factors that can affect breast cancer prognosis. One of these factors is gene copy number variation (CNV)[2,3]. CNV changes have been introduced as an influential feature in human phenotypes and clinical diseases[4,5]. Genome copy number alterations are important factors in tumor development[6]. Since the result of cancer treatment depends on the early diagnosis of the disease, CNV determination in genes involved in cancer may be an effective step in the early detection of breast cancer[7]. Accumulating evidence suggests that breast cancer in patients under 40 is more aggressive and associated with poor outcome than in their older counterparts[8]. As there is an increasing outbreak of the disease in Iran, which is usually detected at advanced stages, the early detection of breast cancer would be helpful in reducing the mortality rate and improving patients’ prognosis[9,10].

Genomic changes, including increase in the expression levels or copy number of those genes which are involved in mitosis, cell cycle progression, embryogenesis, DNA reproduction, cell division, and proliferation, have been shown that are effective in development and progression of breast cancer[11-13].

Baculoviral inhibitor of apoptosis repeat-containing 5 (*BIRC5*) gene, also known as survivin, is a family member of the inhibitors of apoptosis. There are two copies of the aforementioned gene in the entire normal diploid cells that are located on 17q25 chromosome. The *BIRC5* gene product is supposed to play a role in the prevention of apoptosis. It is also known that this gene participates in cell cycle progression and assists the cells to go through the cell cycle checkpoints. This gene has an important function both in tumorigenesis and tumor progression. In a study on pancreatic cancer, *BIRC5* gene amplification has been reported[14]. BIRC5 gene amplification was also observed in lung cancer using multiplex ligation-dependent probe amplification (MLPA) technique[15]. In this regard, *BIRC5* may have the potential to be considered as a therapeutic target in cancer.

With regard to the fact that *BIRC5* is expressed at the embryonic stage but its expression is absent in normal adult tissues, changes in the copy number of this gene can possibly be effective in development and progression of cancer by preventing apoptosis in cells. Hence, this study was conducted to determine the association between *BIRC5* genomic copy number variation and the age of onset in breast cancer.

## MATERIALS AND METHODS

In this study, 40 tumor tissues of breast cancer and 6 normal breast tissues, as multiplex ligation-dependent probe amplification references, were randomly selected and obtained from National Tumor Bank of Iran, Cancer Institute, Imam Khomeini Hospital Complex, Tehran, Iran. Clinicopathological information such as stage, grade, and tumor size of each patient was also obtained from Tumor Bank for further analysis.

### DNA extraction

DNA was extracted from breast tissue samples using the QIAamp DNA mini kit (Qiagen, USA) according to the manufacturer’s instruction. The quality and integrity of DNAs were evaluated by agarose gel electrophoresis. The concentration of high-quality extracted DNA was standardized by using a NanoDrop ND-2000 spectrophotometer (Thermo Scientific, USA).

### Multiplex ligation-dependent probe amplification (MLPA)

The SALSA MLPA P078-C1 Breast Tumor probe kit (MRC, Holland) was used to determine *BIRC5* gene amplification status. In each PCR reaction, three normal DNA samples and one no-template control (NTC) containing TE solution (0.1 mM EDTA+10 mM Tris-Hcl, pH 8.2) were included. All denaturation, hybridization, ligation, and PCR reactions were performed by Peqlab thermocycler (Germany). PCR products were then separated on an ABI3130 capillary sequencer (Applied Biosystems, USA).

### Multiplex ligation-dependent probe amplification analysis

Analysis of *BIRC5* gene copy number was carried out using GeneMarker ver 1.6 (softgenetics, USA). As *BIRC5* has more than one probe in the provided kit, the mean of all the probe peaks of this gene was calculated. If the mean value was below 0.7, the respective gene was defined as lost, while values between 0.7–1.3 and >1.3 were assigned as normal and amplified, respectively[16,17].

### Statistical Analysis

The statistical analyses were carried out by SPSS software package (PASW Statistics for Windows, Version 18.0. Chicago, USA). Significant index was checked by a factor of 95%, and *P*<0.05 was considered to indicate statistical significance.

## RESULTS

**Fig. 1:**
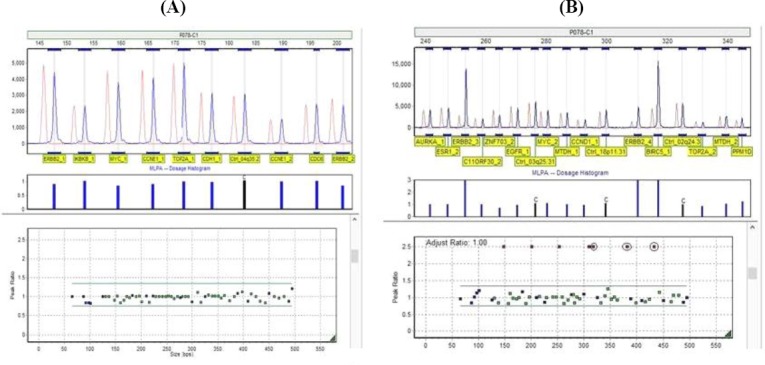
Multiplex ligation-dependent probe amplification (MLPA) analysis of two samples using genemarker software. (A) A breast cancer patient with no genomic copy number changes showing a tumor sample with no changes in BIRC5 genomic copy number. As it is evident, the height of the peaks of the sample (blue) is equal to that of the standard size (orange). (B) A breast cancer patient with BIRC5 gene amplification showing a tumor sample of a breast cancer patient with BIRC5 gene amplification. BIRC5 gene probes are marked with a circle.

In this study, DNA copy number changes in 40 tumor tissues and 6 normal breast tissues were evaluated by using MLPA. Of the total 40 tumor samples, 17.5% (7 cases) showed *BIRC5* amplification. Examples of a breast cancer patient with no genomic copy number changes and a patient with *BIRC5* gene amplification are depicted in Figure 1A and 1B, respectively.

The age range of 40 samples included in this study was between 29 to 77 years, and the average was 50.27 years. Among these patients who showed the *BIRC5* gene amplification, 71% (5 cases) were under 40 years.

Analysis of the *BIRC5* gene amplification and the comparison of changes with the age of onset of the disease showed a significant association between the breast cancer incidence in women under 40 years and an increase in *BIRC5* copy number variation (*P*=0.04). Also, no correlation was found between the clinicopathological characteristics of the patients and *BIRC5* gene amplification status (Table 1).

**Table 1:** Distribution of BIRC5 CNVs in association with clinicopathological characteristics

Clinicopathological characteristics	BIRC5 amplification (Number=7) (%)	*P* value	No BIRC5 amplification (Number=33) (%)	*P* value
Stage				
I	0 (0)	0.6	2 (5)	0.6
II	5 (12.5)		18 (45)	
III	2 (5)		13 (32.5)	
IV	0 (0)		0 (0)	
Grade				
I	2 (5)	0.2	12 (30)	0.5
II	4 (10)		13(32.5)	
III	1 (2.5)		8 (20)	
Age				
≤40	5 (12.5)	0.04	6 (15)	0.3
>40	2 (5)		27 (67)	
Tumor size (cm)				
≤2	0 (0)	0.4	6 (15)	0.6
2<T≤5	5 (12.5)		23 (57)	
>5	2 (5)		4 (10)	

## DISCUSSION

Although cancer is not prevalent in individuals under the age of 40, the incidence of the disease at a younger age may increase the risk of recurrence[18]. Breast cancer in young women is more acute with a poorer prognosis and overall survival in comparison with elder women diagnosed with the disease[11]. Hence, finding those factors that may be related to early onset of breast cancer can lead to faster diagnosis and longer period of disease free survival. *BIRC5* is expressed in human embryonic tissues, while it is not detected in normal adult tissues[19]. The overexpression of this gene in tumor tissues has been already reported [20].

In the present study, the genomic copy number variation of *BIRC5*, which is supposed to be involved in breast cancer progression, was examined, and its association with the early-onset breast cancer was also evaluated. Also,. 17.5% of the patients showed an increase in *BIRC5* gene copy number, and half of the patients under 40 years had amplified *BIRC5* gene. Increase in the *BIRC5* gene copy number in patients who were younger than 40 years showed a significant relationship between the two indices (*P=0.04)*.

Using MLPA technique, Kornegoor *et al*.[21] have studied the genomic copy number alterations for several oncogenes including *BIRC5* in males with breast cancer. They found that among 110 studied samples, 40% of the patients showed an increase in the number of genomic copies of *BIRC5* gene. The differences between our findings and Kornegoor *et al*.[21] results may be due to alterations in patients’ sex, race, and the size of sample population. Moelans *et al*.[22] also used MLPA technique to compare genomic *BIRC5* gene copy number changes in ductal carcinoma *in situ* and invasive ductal carcinoma, in which 20% of the patients with ductal carcinoma *in situ* type breast cancer and 12% of the patients with invasive ductal carcinoma type showed amplified *BIRC5* gene copy number.

Using reverse transcription-PCR technique, Span *et al*.[23] measured mRNA level of this gene in 275 breast cancer patients. They observed a significant relationship between younger patients and *BIRC5* mRNA level in tumor samples. Colak *et al*.[11] also detected genomic changes in *BIRC5* gene among patients under 45 years by studying the whole genome mRNA expression in cancer, from non-invasive breast cancer to invasive breast cancer.

Sought to examine the relationship between the early onset of cancer and increase in the number of *BIRC5* gene copies in tumors, Alaggio *et al*.[24] examined genomic copy number of the gene *BIRC5* using FISH technique in malignant peripheral nerve sheath tumors. They concluded that the number of *BIRC5* gene copies in 35% of the patients in the first two decades of their life was increased.

Baykara *et al*.[15] analyzed the copy number of 22 genes, including *BIRC5*, in 82 tumor tissues of lung cancer. Similar to our results they did not find any possible association with clinical parameters.

By examining the number of gene copies involved in cancer development and treatment, it is possible to determine the cancer progression and the onset age with stronger prognostic. With prospective studies, larger sample sizes, and confirmation of the predictive role between *BIRC5* gene amplification and the early onset of breast cancer, it is possible to use *BIRC5* gene as a marker for cancer detection and the onset age prediction, as well as the likelihood of recurrence of the disease. Moreover, the results of this study indicate that increase in the expression of *BIRC5* gene may not only be due to variation in transcription factors but also may occur along with the increase in the gene copy number. It is also worth mentioning that analyzing gene expression at the mRNA level by real-time PCR or at the protein level by Western blot could help to confirm these results.
